# Clinical validity of increased cortical uptake of [^18^F]flortaucipir on PET as a biomarker for Alzheimer’s disease in the context of a structured 5-phase biomarker development framework

**DOI:** 10.1007/s00259-020-05118-w

**Published:** 2021-02-06

**Authors:** E. E. Wolters, A. Dodich, M. Boccardi, J. Corre, A. Drzezga, O. Hansson, A. Nordberg, G. B. Frisoni, V. Garibotto, R. Ossenkoppele

**Affiliations:** 1 Department of Radiology & Nuclear Medicine, Amsterdam Neuroscience, Vrije Universiteit Amsterdam, Amsterdam UMC, location VUmc, PO Box 7057, 1007 MB Amsterdam, The Netherlands; 2 Alzheimer Center Amsterdam, Department of Neurology, Amsterdam Neuroscience, Vrije Universiteit Amsterdam, Amsterdam UMC, Amsterdam, The Netherlands; 3 NIMTlab - Neuroimaging and Innovative Molecular Tracers Laboratory, University of Geneva, Geneva, Switzerland; 4 Late Translational Dementia Studies Group, German Center for Neurodegenerative Diseases (DZNE), Rostock-Greifswald site, Rostock, Germany; 5 CURIC, Centre Universitaire Romand d’Implants Cochléaires, Department of Clinical Neurosciences, University of Geneva, Geneva, Switzerland; 6 Faculty of Medicine, University of Cologne, Cologne, Germany; 7 Institute of Neuroscience and Medicine (INM-2), Molecular Organization of the Brain, Research Center Jülich, Jülich, Germany; 8 German Center for Neurodegenerative Diseases (DZNE), Bonn-Cologne, Germany; 9 Memory Clinic, Skåne University Hospital, Malmö, Sweden; 10 Clinical Memory Research Unit, Department of Clinical Sciences Malmö, Lund University, Lund, Sweden; 11 Division of Clinical Geriatrics, Center for Alzheimer Research, Department of Neurobiology, Care Sciences and Society, Karolinska Institutet, Stockholm, Sweden; 12 LANVIE - Laboratory of Neuroimaging of Aging, University of Geneva, Geneva, Switzerland; 13 Memory Clinic, University Hospital, Geneva, Switzerland; 14 Centre for Mind/Brain Sciences-CIMeC, University of Trento, Rovereto, Italy

**Keywords:** Alzheimer’s disease, Strategic roadmap, Biomarker-based diagnosis, [^18^F]flortaucipir, PET

## Abstract

**Purpose:**

In 2017, the Geneva Alzheimer’s disease (AD) Biomarker Roadmap initiative adapted the framework of the systematic validation of oncological diagnostic biomarkers to AD biomarkers, with the aim to accelerate their development and implementation in clinical practice. With this work, we assess the maturity of [^18^F]flortaucipir PET and define its research priorities.

**Methods:**

The level of maturity of [^18^F]flortaucipir was assessed based on the AD Biomarker Roadmap. The framework assesses analytical validity (phases 1–2), clinical validity (phases 3–4), and clinical utility (phase 5).

**Results:**

The main aims of phases 1 (rationale for use) and 2 (discriminative ability) have been achieved. [^18^F]Flortaucipir binds with high affinity to paired helical filaments of tau and has favorable kinetic properties and excellent discriminative accuracy for AD. The majority of secondary aims of phase 2 were fully achieved. Multiple studies showed high correlations between ante-mortem [^18^F]flortaucipir PET and post-mortem tau (as assessed by histopathology), and also the effects of covariates on tracer binding are well studied. The aims of phase 3 (early detection ability) were only partially or preliminarily achieved, and the aims of phases 4 and 5 were not achieved.

**Conclusion:**

Current literature provides partial evidence for clinical utility of [^18^F]flortaucipir PET. The aims for phases 1 and 2 were mostly achieved. Phase 3 studies are currently ongoing. Future studies including representative MCI populations and a focus on healthcare outcomes are required to establish full maturity of phases 4 and 5.

**Supplementary Information:**

The online version contains supplementary material available at 10.1007/s00259-020-05118-w.

## Introduction

**Fig. 1:**
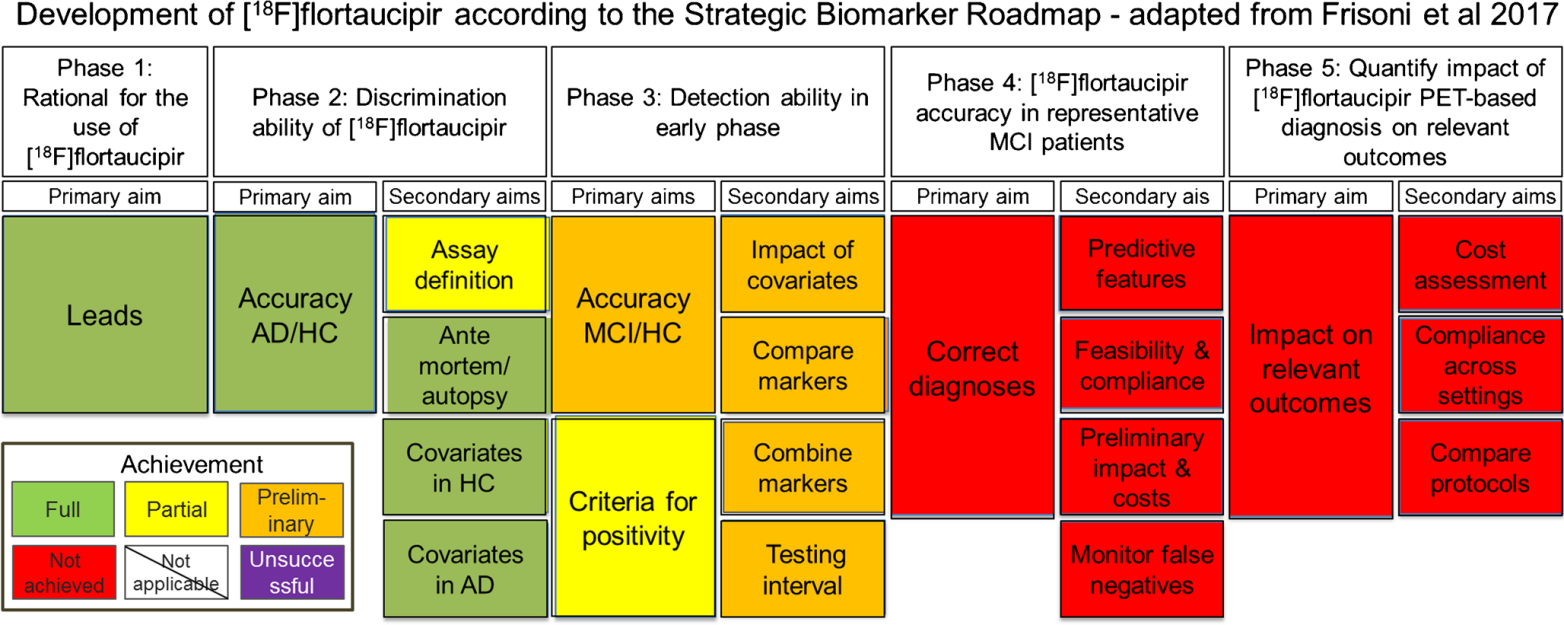
The development of [^18^F]flortaucipir according to the Strategic Biomarker Roadmap

In 2017, a methodological framework for the systematic assessment of biomarker validation was imported from oncology [94] and adapted to Alzheimer’s disease (AD) [10]. This framework assesses analytical validity (phases 1–2), clinical validity (phases 3–4), and clinical utility (phase 5) in steps to be fulfilled sequentially to prevent conveying uncontrollable variability in downstream validation studies (Fig. 1). Within this “Biomarker Roadmap” initiative, we assessed the validation status of consolidated AD biomarkers at that time [30]: episodic memory [14], cerebrospinal fluid (CSF) [78], medial temporal atrophy [111], FDG-PET [32], amyloid PET [16], and 123I-ioflupane brain single-photon emission tomography and 123I-MIBG cardiac scintigraphy [109].

The aim of this work is to assess the validation status of the tau PET tracer [^18^F]flortaucipir based on the Biomarker Roadmap methodology. Tau PET has been recently introduced among the T biomarkers in the AT(N) research framework (A = amyloid-β, T = tau, N = neurodegeneration [47]). Despite the promising preliminary results in the last few years, its maturity for standard use in clinical practice has yet to be defined. We now have developed a methodological framework to assess biomarkers of brain tauopathy [8].

The first-generation tau tracer [^18^F]flortaucipir was first described in 2013 [15, 126] and is currently the most widely used tau PET tracer worldwide. [^18^F]Flortaucipir binds predominantly to paired helical filaments (PHFs) typically observed in AD [29, 68, 75, 126] and was recently approved by the US Food and Drug Administration for detection of aggregated tau pathology by visual read in persons with suspected AD dementia [27]. This review systematically investigates [^18^F]flortaucipir PET studies in order to assess the validation maturity of [^18^F]flortaucipir PET and to define its clinical validity for the diagnosis of (prodromal) AD.

## Methods

### Target

This literature review investigates the validation status of tau PET with [^18^F]flortaucipir as biomarker of neurodegenerative disorders possibly due to AD, in accordance with the 2017 Biomarker Roadmap [10, 30] and its updates [8]. The target population consists of patients with mild cognitive impairment (MCI) referring to memory clinics for ascertained cognitive complaints, attributed to possible sporadic and not familial neurodegenerative disorders leading to dementia. Validation studies of [^18^F]flortaucipir were eligible for this review when including AD neuropathology, in vivo amyloid status as determined by AD biomarkers, or development of incidental AD dementia after 2 years of follow-up as reference standard for the biomarker-based diagnosis. Thus, eligible studies included both prospective longitudinal and cross-sectional studies. This review will only assess the evidence available for [^18^F]flortaucipir. Other tau PET tracers [7, 17] and tau biofluid markers [1, 64] will be discussed elsewhere.

### Glossary

#### Alzheimer’s disease

By Alzheimer’s disease, we refer to the presence of extracellular amyloid-β plaques and aggregates of hyper-phosphorylated tau in neurofibrillary tangles. These features define AD independently of the clinical expression of cognitive symptoms.

#### AD dementia

AD dementia denotes an acquired and progressive cognitive and functional loss of autonomy, according to previous criteria as defined by the National Institute of Neurological and Communicative Disorders and Stroke and the Alzheimer’s disease and Related Disorders Association (NINCDS-ADRDA) criteria [82]. Notably, because of the imperfect accuracy of purely clinical criteria, a percentage of AD dementia cases might have underlying non-AD pathology.

#### Mild cognitive impairment

This refers to a condition within the AD population without functional disability, but with an acquired objective cognitive impairment. Representing a clinical syndrome, it encompasses cases progressing to AD (~ 50%) or non-AD dementia (about 10–15%) [5, 48, 101] as well as stable cases (about 35–40%). MCI cases positive to AD biomarkers have been defined as “prodromal AD” following previous guidelines [26]. The diagnosis of AD at the MCI stage represents the focus of the AD Biomarker Roadmap.

#### Non-AD neurodegenerative disease

This term refers to all neurodegenerative disorders considered for the differential diagnosis, including a large pathological spectrum (hippocampal sclerosis, limbic-predominant age-related TDP-43, frontotemporal lobar degeneration (FTLD), Lewy body dementia (LBD), chronic traumatic encephalopathy, multiple system atrophy, and so forth).

### Conceptual framework

The conceptual framework is described in detail in the Boccardi et al. [10]. The phases and fulfillment of aims were initially developed in oncology [94], adapted to AD [10], and recently updated [8]. This conceptual framework allows for systematic assessment of analytical validity (phases 1–2), clinical validity (phases 3–4), and clinical utility (phase 5) through primary and secondary aims. Analytical validity (i.e., accuracy) of [^18^F]flortaucipir is demonstrated with respect to the gold standard (neuropathology) and is also present when the assay provides measurements with sufficient precision (i.e., reliability), that are consistent over time and in different contexts or circumstances. The clinical validity of [^18^F]flortaucipir is the ability to detect the presence of a sign that is clearly distinct from normal controls, and from “adjacent” signs (or proxies for diseases) on the other hand. Once the biomarker–disease association is established and understood, standard tests to determine the customary validity measures (i.e., sensitivity and specificity) should be conducted to formally explore how the test performs in practice. The clinical utility of [^18^F]flortaucipir is a function of the clinical implications of the results. The purpose of the test is of paramount importance to establish its clinical utility, which can potentially be achieved even though the disease (i.e., MCI due to AD) is not yet fully understood [8, 10].

For each phase/aim, different strings were used to detect relevant studies, which were selected following PRISMA guidelines (see online resource for strings and PRISMA results). For all included studies, relevant information about study design, methods, and results were recorded.

#### Phase 1

This phase assesses analytical validity and includes preclinical exploratory studies on the rationale for using [^18^F]flortaucipir for diagnostic purposes for AD. The gold standard for phase 1 studies is neuropathology.

#### Phase 2

Phase 2 studies, still entailing analytical validity, investigate the diagnostic accuracy of [^18^F]flortaucipir to distinguish patients with AD dementia from controls. Phase 2 studies are meant to define the clinical assay to allow reliable assessment and identify the effect of confounders on the level of biomarker that may affect the threshold for positivity in both patients and controls (e.g., age, gender, apolipoprotein ε4 (APOE ɛ4) status, education, or comorbidities).

#### Phase 3

Phase 3 studies assess clinical validity, i.e., the ability of the biomarker to detect the disease at its earliest possible phase, namely MCI for this specific effort, in well-controlled experimental samples. Phase 3 studies aim to define criteria for positivity, to compare the diagnostic performance with other biomarkers, and to assess the diagnostic value of combinations of biomarkers, in view of defining a biomarker-based algorithm.

#### Phase 4

Phase 4 studies assess the clinical validity of [^18^F]flortaucipir in representative patient cohorts from memory clinics. The biomarker itself is used to deliver a clinical diagnosis to patients with MCI who are subsequently treated based on this biomarker-based diagnosis. They are meant to ascertain clinical validity in patients with comorbidities and less strictly controlled conditions, and to start quantify the benefit of biomarker-based early detection, practical feasibility, protocol compliance, and costs to prepare phase 5.

#### Phase 5

Phase 5 studies quantify the clinical utility of [^18^F]flortaucipir-based diagnosis in terms of impact on society (e.g., cost-effectiveness relative to clinically meaningful outcomes).

### Assessment of aim compliance

The fulfillment of each validation step from phase 1 to phase 5 has been assessed consistently with the 2017 Biomarker Roadmap and the methodological update [8, 10]. However, in this initiative, we have performed a data extraction that summarizes the available data, thus allowing the reader to make its own appraisal of aim compliance and preparing to sounder evidence assessment. To that end, for each primary and secondary aim of each study, we have extracted data consistent with formal evidence assessment as previously described [9]. Tables with data extraction are accessible online (https://drive.switch.ch/index.php/s/4reUTSuqNZHyIC8).

Potential outcomes for each aim include:

1. Fully achieved: available scientific evidence, successfully replicated in properly powered and well-designed studies.
2. Partly achieved: the available evidence is not sufficiently replicated, or samples are not adequately powered, or studies are faulted with major methodological limitations.
3. Preliminary evidence: only preliminary evidence is available.
4. Not achieved: studies are not yet performed at the time of the review.
5. Unsuccessful: Available scientific evidence shows a failure for the biomarker in achieving the aim. Findings in the subsequent roadmap phases should be interpreted with caution.

### Manuscript search and selection

PubMed and Embase® were searched for relevant studies. The search was conducted on 05.05.2020 by author EW and replicated by author JC.

The keywords used to identify articles about [^18^F]flortaucipir (formerly known as AV1451 or T807) PET imaging are reported in supplementary Table 1.

We first screened the title and abstract of the papers, added papers from other sources (personal knowledge, references from these or other papers), and then excluded redundancies. The reasons for exclusion and the number of finally retained papers are reported according to the PRISMA guidance. Details for each phase/aim are available on online resource.

## Results

### Current clinical validity of tau PET imaging

#### Phase 1. Preclinical exploratory studies

##### Phase 1. Primary aim

To identify and prioritize leads for potentially useful biomarkers.

Neurofibrillary tau tangles are one of the main pathological hallmarks of AD [11, 37, 45]. [^18^F]Flortaucipir binds to PHFs of tau with a 25-fold higher affinity than for amyloid-β in AD patients [15, 68, 75, 126]. However, the tracer is also characterized by off-target binding in the basal ganglia, thalamus, and choroid plexus [68, 75]. The in vivo kinetics of [^18^F]flortaucipir are described as favorable, with rapid clearance from plasma and polar metabolites not entering the brain [3, 4, 34, 39, 125]. This aim was considered fully achieved (Fig. 1).

#### Phase 2. Clinical assay development for clinical Alzheimer’s disease

##### Phase 2. Primary aim

To estimate true positive and false positive rates, or receiving operating characteristics curves (ROC) for the essay and to identify the discrimination accuracy between subjects with and without the disease.

To date, one multi-center study comprising 719 participants assessed the diagnostic accuracy of [^18^F]flortaucipir PET in distinguishing AD from non-AD neurodegenerative disorders [90]. The gold standard was a clinical diagnosis of AD supported by amyloid-β-positive biomarkers. The area under the curves (AUCs) of [^18^F]flortaucipir uptake in the medial basal and lateral temporal cortex were 0.94–0.98, depending on the cutoff methods used for distinguishing AD dementia from non-AD neurodegenerative disorders. Similar results were found in another study [52]. The discriminative accuracy was lower for MCI due to AD vs. non-AD neurodegenerative diseases with an AUC of 0.82 [90]. In a secondary analysis, the diagnostic performance of [^18^F]flortaucipir PET in distinguishing MCI due to AD (AUC 0.86)/AD dementia (AUC 0.97) vs. controls was examined. In addition, two other studies investigated the diagnostic performance of [^18^F]flortaucipir PET in a clinical sample, which consisted of both AD and non-AD neurodegenerative disorders [60] and prodromal/AD dementia and controls [77, 81]. However, both cohorts included overlapping samples with the earlier described larger multi-center study [90]; therefore, we do not consider these results independently. Another study assessed partly a new cohort in AD neuroimaging (ADNI), consisting of MCI/AD patients and Aβ older controls. The diagnostic performance of [^18^F]flortaucipir for distinguishing MCI/AD from controls was overall lower compared to previous study [90] with AUC values between 0.76 and 0.87 [71]. In addition, when [^18^F]flortaucipir hippocampal and AD cortical signature regions were used for distinguishing AD from controls, AUCs of 0.89 to 0.98 were found, respectively [119]. This aim was considered fully achieved (Fig. 1).

##### Phase 2. Secondary aim 1

To optimize procedures for performing the assay and to assess its reproducibility within/between laboratories.

The radio synthesis and purification of [^18^F]flortaucipir were optimized by using fully automatic procedures with less hazardous solvents and radiotracer doses which are applicable for clinical use [31, 44, 86, 106]. The semi-quantitative standardized uptake value ratios (SUVr) of the most widely used time window of 80 to 100 min post-injection correlated reasonably well with fully quantitative methods in cross-sectional studies [3, 4, 28, 34, 39, 42, 125].

To test the reliability of [^18^F]flortaucipir, test–retest (TRT) studies have been performed. In general, these studies show excellent TRT reproducibility [24, 114]. For SUVr_80–100 min_, values of the percentage of change ranged between 1.5 and 3.3% [114] and 0.7 and 4.3% depending on the reference region and regions of interest. Quantitative methods (TRT ≈ 2%) performed slightly better than semi-quantitative measures such as SUVr (TRT ≈ 3%) [115]. Recently, guidelines for visual interpretation of [^18^F]flortaucipir images have been developed [29]. This was based on visual [^18^F]flortaucipir assessments performed by five readers that yielded high accuracy (~ 0.88) for assessing advanced tau stages (Braak V or VI) [29]. More specific guidelines and training reader programs for nuclear medicine specialists have yet to be developed. This aim is considered partly achieved (Fig. 1).

##### Phase 2. Secondary aim 2

To determine the relationship between biomarker measurements made on brain tissue and the biomarker measurements made on the non-invasive clinical specimen.

Autopsy studies with ante-mortem [^18^F]flortaucipir scans combined with post-mortem pathology showed strong associations between in vivo [^18^F]flortaucipir uptake and the amount of post-mortem tangles with rhos varying from 0.61–0.93 [29, 69, 108]. Importantly, these strong associations were found for AD-like tau pathology and not for non-AD tau aggregates [74]. Elevated in vivo [^18^F]flortaucipir uptake was predominantly observed in Braak IV or higher [29, 69]. Braak V and higher was detected with a sensitivity ranging from 92.3 (95% CI, 79.7–97.3%) to 100.0% (95% CI, 91.0–100.0%) and specificity ranging from 52.0 (95% CI, 33.5–70.0%) to 92.0% (95% CI, 75.0–97.8%) [29]. This aim is considered fully achieved (Fig. 1).

##### Phase 2. Secondary aim 3

To assess factors (e.g., sex, age) associated with biomarker status or level in control subjects.

In cognitively normal elderly, [^18^F]flortaucipir uptake is typically mostly confined to the medial temporal lobe (MTL) [56, 97, 102, 116]. The presence of amyloid-β may induce tau to spread outside of the MTL [53, 128], although neocortical tau was present in amyloid-negative controls [67, 120]. Both cross-sectional [56, 66, 70, 85, 95, 100, 105, 110, 119, 128] and antecedent amyloid accumulation [62, 116] were correlated with more (extra-)MTL [^18^F]flortaucipir in the cognitively unimpaired. In addition, longitudinal [^18^F]flortaucipir data also showed that an antecedent rise of amyloid-β was associated with a subsequent rise of tau accumulation in the inferior temporal lobe [40]. Recent studies found greater rates of tau accumulation (~ + 0.5% SUVr/year) in amyloid-positive vs. amyloid-negative control subjects [49, 96]. However, another study observed accumulation of tau at similar rates for amyloid + vs. − cognitively normal individuals [41].

Two studies showed that APOE ɛ4 carriers had increased levels of entorhinal [^18^F]flortaucipir retention; however, these effects were largely attributable to elevated amyloid-β levels [33, 100], while studies in cognitively unimpaired controls using ADNI data showed that APOE ɛ4 was associated with increased [^18^F]flortaucipir uptake in the MTL, independently of amyloid burden [112, 121]. Furthermore, a study in healthy controls (41.2% Aβ+) found higher tau SUVrs in the parahippocampal gyrus in ɛ3ɛ3 carriers compared to ɛ2ɛ3 carriers, after adjusting for amyloid. This potentially shows the protective effect of the ɛ2 allele, although this must be interpreted with caution since the number of ɛ2ɛ3 carriers was limited (*n* = 11) [95].

The influence of sex on the amount of tau pathology in controls has yet to be determined, but mounting evidence is provided towards the conception that women harbor more tau pathology than men. One study in two independent cohorts of cognitively normal subjects found that in the presence of high amyloid burden, women had higher entorhinal tau load than man [13]. This observation was confirmed in a study showing higher tau retention in temporo-parietal and frontal areas in women [95]. Another study suggested that men have higher uptake mainly in the frontal and parietal white matter and thalamus than women [128], although this was hypothesized to be largely driven by non-specific binding.

Few studies have investigated the association between cardiovascular risk factors/ small vessel disease and the amount of [^18^F]flortaucipir retention. Higher cardiovascular risk score was related to higher tau uptake in temporal neocortical regions, in the presence of high amyloid-β burden [99]. When examining the separate components of the risk score, it was found that body mass index, treatment with antihypertensive medication, systolic blood pressure and smoking status all significantly contributed to this effect [99]. Another study including controls with a positive family history for sporadic AD found no effect of vascular risk factors on entorhinal tau burden [58]. A large study in 434 controls did not find an association between white mater hyperintensities on MRI and increased [^18^F]flortaucipir retention [36].

Higher age is associated with higher [^18^F]flortaucipir uptake in the temporal lobe [83, 110], even independently of amyloid status [67, 72]. The observation of [^18^F]flortaucipir uptake in the MTL in the absence of widespread neocortical amyloid plaques has been referred to as primary age-related tauopathy (PART) [21]. PART is a neuropathological description of the presence of NFTs in the MTL, basal forebrain, and olfactory areas, without abundant amyloid-β pathology. Interestingly, both neuropathological studies [12, 98] and [^18^F]flortaucipir PET studies [19, 41, 49] indicate that NFTs may not consistently spread outside of these areas without amyloid-β. Therefore, it could be argued that [^18^F]flortaucipir PET uptake in the MTL in the absence of amyloid-β is an age-related phenomenon and amyloid-β is necessary to trigger the spread of tau pathology.

African American ethnicity may be associated with higher [^18^F]flortaucipir uptake. One smaller study demonstrated higher [^18^F]flortaucipir SUVrs in the hippocampus and choroid plexus in the Black/African American population when compared to White participants [63]. These differences may be related to off-target binding to melanocytes in the choroid plexus causing spill-in into the hippocampus, since no differences were found in other regions of interest (ROIs). This is corroborated by another study which found that Black race was associated with higher [^18^F]flortaucipir retention in occipital, temporal, and frontal clusters closely to meninges, which is known to contain high levels of neuromelanin [128].

A study in 325 individuals, mostly (90%) consisting of cognitively impaired controls, found no effect of education on the amount of [^18^F]flortaucipir retention [100].

This aim is considered fully achieved (Fig. 1).

##### Phase 2. Secondary aim 4

To assess factors associated with biomarker status or level in cognitively impaired subjects—in particular, disease characteristics such as stage, molecular features, and prognosis.

There is a positive association between the level of cerebral amyloid load with greater [^18^F]flortaucipir uptake in the brain [22, 56, 71, 87, 93, 97, 119, 122]. This is corroborated by longitudinal studies indicating that antecedent amyloid accumulation/status is predictive of higher rates of tau accumulation over time [19, 47, 96, 116]. Younger AD patients display higher levels of neocortical [^18^F]flortaucipir uptake than older patients [20, 59, 67, 92, 103, 116, 123], while older age is associated with greater [^18^F]flortaucipir uptake specifically in the medial temporal lobe [92, 116, 122].

Studies comprising cognitively normal and patients with MCI due to AD [116] and MCI due to AD and AD dementia [88] did not observe sex differences in [^18^F]flortaucipir uptake.

Studies focusing on APOE genotype have reported conflicting results in how APOE genotype impacts the amount of [^18^F]flortaucipir uptake in the brain. Two studies showed that amyloid+ APOE ɛ4–negative carriers had higher [^18^F]flortaucipir uptake in neocortical areas compared their APOE ɛ4–positive counterparts [79, 123]. In a smaller study comprising various AD patients with non-amnestic presentations, APOE ɛ4 carriers showed greater temporal and parietal [^18^F]flortaucipir uptake [92]. Others found no association between APOE ɛ4 status and [^18^F]flortaucipir uptake [56, 116]. A larger study in 108 cognitively impaired patients found that APOE ɛ4 was associated with increased tau PET uptake in the entorhinal cortex [112]. In addition, women seem to be more susceptible to APOE ɛ4–associated accumulation of neurofibrillary tangles in MCI compared to males, although this effect was only observed in non-partial volume corrected data [65].

To date, years of education was not associated with [^18^F]flortaucipir uptake in some studies largely including MCI due to AD patients [56, 116]. A study including 24 patients with AD dementia showed that higher education was associated with higher [^18^F]flortaucipir retention in more advanced Braak stages [43]. This aim is considered fully achieved (Fig. 1).

#### Phase 3. Retrospective/prospective/longitudinal repository studies

##### Phase 3. Primary aim 1

To evaluate, as a function of time in the prodromal stage (MCI), the capacity of the biomarker to predict conversion to AD dementia.

Few cross-sectional studies distinguished MCI due to AD from non-AD [50, 90]. AUCs ranging from 0.82 to 0.86 were found for distinguishing MCI due to AD from non-AD neurodegenerative diseases or controls. Since MCI due to AD is very likely to progress to AD, this provides preliminary evidence for the usefulness of [^18^F]flortaucipir for predicting conversion to AD dementia.

Although not within the scope of this review (which is aimed at the prodromal phase of AD), note that a study in cognitively normal older adults showed that tau accumulation was associated with progression from preclinical AD to MCI [40]. Importantly, the amount of amyloid accumulation did not differ between the progressors (*n* = 6) and stable (*n* = 11) participants.

To date, there are no longitudinal studies available which predict the conversion of MCI patients to AD dementia. Since only cross-sectional data is available, this aim is considered preliminarily achieved.

##### Phase 3. Primary aim 2

Define criteria for a positive diagnostic test for MCI due to AD, in preparation of phase 4.

Determining tau positivity requires careful selection of brain regions characterized by [^18^F]flortaucipir uptake for defining an appropriate cut point. Various methods have been suggested, including approaches that recapitulate the neuropathological defined Braak stages [71, 102, 104] as well as different regional and global qualitative measures [51, 71, 85, 90, 119, 120]. The final selection may depend on the clinical question at stake (e.g., early detection, differential diagnosis, tracking disease progression over time). The jury is not yet out, but entorhinal cortex, inferior temporal cortex, a temporal meta-ROI (consisting of the entorhinal, amygdala, parahippocampal, fusiform, inferior temporal, and middle temporal ROI), temporo-parietal cortex, whole cortex, and possibly data-driven ROIs are among the composite regions that are likely candidates for determination of tau PET positivity [51, 56, 71, 85, 97, 102, 117, 118]. Some of these composite regions show a remarkable consistency across different studies, even though variability in image (pre)processing and acquisition exists, which bodies well for the potential future clinical application of the tracer. A good example of this high consistency is the temporal meta-ROI, showing comparable SUVr cutoffs across studies (1.2–1.4) [51, 69, 71, 85, 90, 119]. Regions involved earlier in AD, such as Braak stages I–II or the inferior temporal lobe, may be more sensitive to detect prodromal AD [19, 40, 71]. This is corroborated by longitudinal study supporting the temporal order of Braak staging with [^18^F]flortaucipir PET, in which uptake rose sequentially from Braak I–II through III–IV to V–VI [2]. To date, there are no studies on visual assessment for solely MCI due to AD yet. However, two studies comprising of largely AD dementia patients investigated the relationship between [^18^F]flortaucipir retention and pathological tau burden and found that a minimum neuropathological Braak stage of IV was necessary to visually detect an elevated AD [^18^F]flortaucipir PET signal [29, 69]. Furthermore, an optimal threshold of 1.29 for the temporal meta-ROI was established to identify a diagnosis of the AD spectrum with a sensitivity and specificity of 87% and 82%, respectively [69]. This aim is considered partly achieved (Fig. 1).

##### Phase 3. Secondary aim 1

To explore the impact of relevant covariates on the biomarker discrimination abilities before the clinical diagnosis.

To date, there are no studies which investigated the influence of certain factors on the diagnostic performance of [^18^F]flortaucipir PET in MCI patients. However, regional tau differences are dependent on age [51] and clinical stage [19], so we may have to use different cutoffs in different populations. Therefore, this aim was considered preliminary at the time of inclusion stop for this review (Fig. 1).

##### Phase 3. Secondary aim 2

To compare biomarkers with a view to selecting those that are most promising.

Regional patterns of [^18^F]flortaucipir show close correspondence to hypometabolic patterns on [^18^F]FDG-PET [6, 25, 91, 92]. Similarly, several studies demonstrated strong anatomical overlap between tau pathology and brain atrophy [18, 23, 46, 57, 61, 73, 87, 114, 119, 123, 127] in MCI and AD patients. In prodromal AD, tau PET was slightly stronger associated with lower scores on cognitive tests than amyloid PET and cortical thickness, suggesting that tau PET is more sensitive than amyloid PET/cortical thickness in measuring cognitive changes early in the disease [93]. Two studies compared tau PET with MRI atrophy measures in order to predict the diagnosis of AD [93]. For both the diagnosis of MCI/AD dementia vs. cognitively unimpaired subjects [77] and vs. non-AD neurodegenerative disorders [90], [^18^F]flortaucipir (AUCs > 0.9) outperformed established MRI measurements such as hippocampal volumes (AUC of ~ 0.6), AD signature cortical thickness (AUCs of ~ 0.8), or whole-brain cortical thickness (AUC of ~ 0.5). To date, no studies have compared the predictive value of these different imaging modalities for the conversion from MCI to AD dementia.

Several cross-sectional studies compared CSF tau biomarkers with [^18^F]flortaucipir tau PET [35, 55, 60, 79, 80, 84, 87, 124]. Two studies compared the diagnostic accuracy for phosphorylated tau (p-tau), total tau (t-tau), and [^18^F]flortaucipir in distinguishing MCI/AD dementia vs. cognitively unimpaired [81] or non-AD neurodegenerative disease [60]. A [^18^F]flortaucipir temporal meta-ROI was better in distinguishing AD dementia from controls (AUC 1.0 vs. t-tau, AUC 0.88; p-tau, AUC 0.89), but all tau biomarkers performed equally well in distinguishing MCI from cognitively normal ([^18^F]flortaucipir, AUC 0.92; t-tau, AUC 0.86; p-tau, AUC 0.94) [79]. Comparable excellent classification was also seen for [^18^F]flortaucipir and CSF p-tau for the differential diagnosis AD vs. non-AD dementias (AUCs 0.92–0.94) [60]. It is important to note that CSF tau biomarkers and [^18^F]flortaucipir PET probably reflect different aspects of tau pathology, which become apparent in the temporal difference of “becoming abnormal” between the biomarkers. That is, CSF p-tau probably changes early in the disease course, and plateaus in early AD [76, 78, 84, 124], while [^18^F]flortaucipir PET likely becomes abnormal after CSF tau biomarkers [76] and continues to increase over time with advancing disease stage [2, 19, 40, 41, 47, 96].

Emerging evidence demonstrated that binary classifications as well as continuous levels of plasma tau phosphorylated at threonine 181 (p-tau_181_) are strongly associated with [^18^F]flortaucipir retention [54, 113]. Furthermore, plasma p-tau_181_ accurately discriminated AD dementia from a variety non-AD neurodegenerative disorders (for example from FTLD or a variety of non-AD disorders with AUCs of 0.89 and 0.93, respectively) [54, 113], although slightly worse than [^18^F]flortaucipir PET (AUC of 0.98) [55].

Currently, there are no studies available that compare the ability of these biomarkers to identify those MCI subsequently progressing to AD dementia. Therefore, this aim was preliminarily achieved (Fig. 1).

##### Phase 3. Secondary aim 3

To develop algorithms for the biomarker-based diagnosis of MCI in preparation of phase 4.

There is no study proposing an algorithm combining [^18^F]flortaucipir to other biomarkers to predict cognitive decline in MCI. A longitudinal study among older persons without dementia at baseline found that a model combining input from amyloid PET, [^18^F]flortaucipir PET, and MRI cortical thickness data provided the most optimal prediction of memory decline [52]. The evidence for this aim is considered preliminary (Fig. 1).

##### Phase 3. Secondary aim 4

To determine an interval able to detect a meaningful change of biomarker status or level in progressing MCI.

Few studies [2, 19, 40, 41, 49, 96] have investigated [^18^F]flortaucipir uptake longitudinally with a maximum time interval of 2 years. Results were mixed and potentially affected by methodological decisions regarding the choice of reference region, regions of interest, and partial volume correction methods. In MCI patients, the patterns of MCI patients progressing to AD differed from the stable MCI subjects during a follow-up period of 2 years [19]. Progressors showed an increase in all cortical regions, except for the sensorimotor cortex, while the cognitively stable participants showed increases in the inferior temporal cortex. Another longitudinal study (with partially overlapping participants from Cho et al. [19]) showed that the annual change in tau accumulation within all Braak regions was intermediate in MCI patients relative to cognitively unimpaired and dementia patients [2]. There is no notion of clinical progression of the MCI patients included in this study.

Other studies did not show results of MCI patients separately from participants with AD dementia [41, 47], but differences were observed in rate of accumulation in amyloid-positive cognitively impaired (+ 3–5% SUVr/year) vs. unimpaired (+ 0.5–3% SUVR/year) subjects in a meta-ROI comprising AD-specific areas of the temporal cortex [41, 49]. Consistently with the requirement that the proper achievement of the downstream validation steps depends on the full achievement of the abovementioned steps, the validation of [^18^F]flortaucipir did not yet enter the validation phases 4–5. This aim was preliminarily achieved (Fig. 1).

## Discussion

With this work, we assessed the maturity of [^18^F]flortaucipir as a biomarker of brain tauopathy according to the 5-phase framework, which was originally developed for oncology biomarkers [94]. We adapted this framework to study populations including MCI-due-to-AD and AD dementia [10], and used it to critically evaluate for which validation steps sufficient evidence has been provided in the literature and to identify the validation steps that require additional research.

We considered phase 1 fully achieved based on (pre)clinical studies that demonstrated the rationale for using [^18^F]flortaucipir. [^18^F]Flortaucipir binds with high affinity to AD PHFs of tau [15, 68, 75, 126], and the in vivo kinetics of [^18^F]flortaucipir are favorable [3, 4, 34, 39, 125]. The primary aim of phase 2 was also considered fully achieved. A large multi-center study found an excellent diagnostic accuracy (AUC = 0.97) of [^18^F]flortaucipir to distinguish patients with AD dementia from controls [90]. Moreover, the test–retest reliability of [^18^F]flortaucipir was excellent, with percentages of change ranging from ~ 1 to 4% [24, 114]. For the secondary aims of phase 2, ante-mortem [^18^F]flortaucipir was strongly associated with post-mortem tau burden [29, 69]. Multiple studies investigated the effect of confounders, such as age, sex, APOE, education, and vascular risk factors on the amount of [^18^F]flortaucipir in both controls and AD patients. Therefore, the majority of the secondary aims of phase 2 are fully achieved. Phase 3 first primary aim was preliminarily achieved, and the secondary primary aim was partly achieved. Only few longitudinal studies in MCI patients are available, and defining tau PET positivity is challenging because many factors (e.g., ROI definition, demographic variables, and disease severity) can impact the threshold. Nevertheless, encouraging results were obtained as studies in multiple independent cohorts have shown that despite the substantial variation in image (pre)processing and acquisition, quantitative cutoffs for a temporal composite ROI were largely comparable [51, 71, 85, 90, 119]. The secondary aims of phase 3 (i.e., comparison between or combining different biomarkers) were preliminarily achieved, because ability of these biomarkers to accurately detect those MCI progressing to AD at follow-up was not determined. Although the accumulation of tau is probably clinically meaningful [19, 40, 47, 96], only preliminary evidence is available to determine the optimal interval for repeating [^18^F]flortaucipir PET scans over time. The aims of phases 4 and 5 (i.e., prospective diagnostic studies and disease-control studies) were not achieved. This kind of work is necessary to coordinate efforts across independent research groups. Greater awareness of completed steps, research gaps, and priorities based on a sound consensual methodological framework guarantees the cost-effectiveness and boosting of the validation procedure.

Our analysis identified at least four areas of research that require further investigation to reach full maturity for [^18^F]flortaucipir PET as a biomarker for brain tauopathy. First, procedures and criteria for [^18^F]flortaucipir PET positivity need to be refined and compared against other (established) biomarkers of AD. The proposed visual read metric for [^18^F]flortaucipir PET [29] has shown to benefit from a complementary quantitative cutoff that reduces the number of false positive cases. It is possible that different thresholds are required, as there is substantial regional variability in the accumulation of tau. For example, visual assessment of early to intermediate tau-specific regions such as Braak stages I–II or the inferior temporal lobe may be challenging, as previous studies showed that a positive visual read was associated with tau pathology in Braak stage IV or higher [29, 69]. Furthermore, not all AD patients adhere to the stereotypical spread of tau pathology as proposed by neuropathological studies [11], as a substantial proportion of AD present with a neocortical-predominant and hippocampal-sparing type of AD [89, 107]. For the comparison with other tau biomarkers, mounting evidence so far points into the direction that CSF p-tau may be more sensitive in detecting tau pathology in the earliest clinical phases of AD [76, 80, 84, 124], although diagnostic accuracy to discriminate MCI patients showed comparable results [81]. At the dementia stage, contrary to CSF p-tau, [^18^F]flortaucipir PET has not yet reached a plateau in the neocortex [2, 19, 40, 41, 47, 96] and can therefore more accurately track disease progression. In addition, compared to tau biofluid biomarkers, [^18^F]flortaucipir PET has the advantage to regionally assess the extent of tau pathology.

A second gap to be filled as research priority is to assess the influence of covariates on determination of [^18^F]flortaucipir positivity. Many studies identified modifiers of tau accumulation in controls, including higher age [70, 72, 83, 103, 110], baseline and longitudinal change in amyloid burden [40, 49, 56, 62, 66, 70, 85, 95, 96, 100, 105, 110, 116, 119, 128], female sex [13, 95], and APOE ɛ4 status [112]. In AD patients, lower age was associated with a higher neocortical tau burden [20, 59, 70, 92, 103, 116, 122], whereas higher age was associated with higher [^18^F]flortaucipir in the medial temporal lobe [92, 116, 123]. Future studies are needed to assess whether flexible [^18^F]flortaucipir positivity thresholds or target regions of interest should be implemented based on patient-specific demographic, clinical, or genetic information.

Finally, there is a clear need for studies that prospectively assess whether [^18^F]flortaucipir PET impacts patients management, healthcare outcomes, and costs, as well as its feasibility in a clinical setting.

This work has some limitations. First, although adhering to sound methodology, the fulfillment of each aim should be based on a more thorough evidence assessment examining many possible sources of bias (e.g., Guyatt et al. [38] “GRADE guidelines: 1. Introduction-GRADE evidence profiles and summary of findings tables”). Our online tables (https://drive.switch.ch/index.php/s/4reUTSuqNZHyIC8) are meant to help this development as a next step forward in a systematic assessment of the validation of AD biomarkers. Second, for the fulfillment of phases 1 and 2, the gold standard of neuropathology is required. AD tissue in combination with ante-mortem imaging data is much less accessible than for example in oncology, the disease for which the original Geneva Roadmap was developed [94]. It is important to note that we also considered feasibility issues when assessing the maturity of the different aims. Third, [^18^F]flortaucipir is situated in a dynamic field of research characterized by rapid development and progression. When interpreting the analysis presented here, one should note that our inclusion stop for published studies was May 5th 2020 and that more validation steps within framework might have been (more) complete(d) in the near future.

## Conclusion

This review systematically investigated [^18^F]flortaucipir PET studies in order to assess the validation maturity of [^18^F]flortaucipir PET and define its clinical validity for the diagnosis of AD. Current literature provides partial evidence for clinical utility of [^18^F]flortaucipir PET. The aims for phases 1 and 2 were largely achieved. In vivo [^18^F]flortaucipir PET shows excellent diagnostic accuracy for AD and promising results for the validation with autopsy studies. Phase 3 studies are currently ongoing. Further studies in phases 4 and 5 including representative MCI populations and focusing on healthcare outcomes are required to establish full maturity.

## Supplementary information

ESM 1 (DOCX 19 kb)
