# Supplementary material for: Clinical validity of increased cortical uptake of [18F]flortaucipir on PET as a biomarker for Alzheimer’s disease in the context of a structured 5-phase biomarker development framework
Source: Eur J Nucl Med Mol Imaging. 2021 Feb 6;48(7):2097–109. doi: 10.1007/s00259-020-05118-w (PMC8175307; doi:10.1007/s00259-020-05118-w)
Supplement: Supplementary file 1 — (DOCX 19 kb) [file 259_2020_5118_MOESM1_ESM.docx]

**Supplementary table 1.** Strings used as reference for the harmonized literature searches of the Reviews on the Maturity of Biomarkers of Mild cognitive impairment due to Alzheimer’s disease.

| Ph |  | **Aim-specific key words string** |
| --- | --- | --- |
| **Phase 1: preclinical exploratory studies** | **Primary aim:** To identify leads for potentially useful biomarkers and prioritize identified leads. | (e) |
| **Phase 2: clinical assay development for clinical disease** | **Primary aim:** To estimate TPR and FPR or ROC curve for the assay and to assess its ability to distinguish subjects with and without disease. | ("accuracy" OR "sensitivity" OR "Specificity" OR "ROC" OR "predictive value") AND (a) AND (b) AND (e) |
|  | **Secondary aim 1:** To optimize procedures for performing the assay and to assess the reproducibility of the assay within and between laboratories. | (“standardization” OR “visual” OR “measure” OR “assessment” OR “reading” OR “quantification”) AND (“reproducibility OR “reliability” OR “agreement”) AND (“Alzheimer”) AND (a) AND (e) |
|  | **Secondary aim 2:** To determine the relationship between biomarker tissue measurements made on tissue (phase 1) and the biomarker measurements made on the noninvasive clinical specimen (phase 2). | (“autopsy” OR “autoptic” OR “pathology” OR “neuropatholog*” OR “histopathol*”) AND (a) AND (e) |
|  | **Secondary aim 3:** To assess factors (e.g. sex, age, etc.), associated with biomarker status or level in control subjects. If such factors affect the biomarker, thresholds for test positivity may need to be defined separately for target subpopulations. | (“effect” OR “association” OR “covariates”) AND (“factor” OR “habit” OR “age” OR “sex” OR “gender” OR “education” OR “life-style” OR “risk factor”) AND (b) AND (e) |
|  | **Secondary aim 4:** To assess factors associated with biomarker status or level in diseased subjects—in particular, disease characteristics. | (“effect” OR “association” OR “covariates”) AND (“factor” OR “habit*” OR “age” OR “sex” OR “gender” OR “education” OR “life-style” OR “risk factor*”) AND (a) OR (c) AND (e) |
| **Phase 3: Prospective repository studies** | **Primary aim 1:** To evaluate the capacity of biomarkers to detect pre-clinical disease and define criteria for a positive biomarker test in preparation for phase 4. | ("follow-up" OR "followup" OR "conversion" OR "progression" OR "decline" OR "predict") AND (c) AND (e) |
|  | **Primary aim 2::** | ("cut-off" OR "cut-point" OR "measure" OR "assessment") and (e) |
|  | **Secondary aim 1:**To explore the impact of covariates on the discriminatory abilities of the biomarker before clinical diagnosis. | (“effect” OR “association” OR “covariates”) AND (“factor" OR “habit” OR “age” OR "sex" OR "gender" OR "education" OR "life-style" OR "risk factor") AND ((a) OR (b) OR (c)) AND (e) |
|  | **Secondary aim 2:** To compare markers with a view to selecting those that are most promising. | ("follow-up” OR “followup” OR “conversion” OR “progression” OR “decline” OR "predict" OR "cut-off" OR "cut-point" OR "measure" OR "assessment") AND ("combinat*" OR "associat*" OR "compar*") AND (a) AND (c) AND (e) |
|  | **Secondary aim 3:**To develop algorithms for positivity based on combinations of markers. | ("follow-up” OR “followup” OR “conversion” OR “progression” OR “decline” OR "predict" OR "cut-off" OR "cut-point" OR "measure" OR "assessment") AND ("combinat*" OR "associat*" OR "compar*") AND (a) AND (c) AND (e) |
|  | **Secondary aim 4:**To determine a biomarker testing interval for phase 4 if repeated testing is of interest. | ("follow-up” OR “followup” OR “conversion” OR “progression” OR “decline” OR "predict" OR "cut-off" OR "cut-point" OR "measure" OR "assessment") AND ("combinat" OR "associat" OR "compar") AND (a) AND (c) AND (e) |
| **Phase 4: Prospective Diagnostic Studies** | **Primary aim:** To determine the operating characteristics of the biomarker-based test in a relevant population by determining the detection rate and the false referral rate. Studies at this stage involve testing people and lead to diagnosis and treatment. | (“diagnosis” OR “treatment”) AND (a) AND (c) AND (e) |
|  | **Secondary aim 1:** To describe the characteristics of disease detected by the biomarker test—in particular, with regard to the potential benefit incurred by early detection. | (“clinical diagnosis” OR “treatment” OR “memory clinic”) AND (“benefits"OR “outcome” OR “improve”) AND (a) AND (c) AND (e) |
|  | **Secondary aim 2:** To assess the practical feasibility of implementing the case finding program and compliance of test-positive subjects with work-up and treatment recommendations. | ("clinical diagnosis” OR “treatment” OR “memory clinic”) AND ("benefit" OR "compliance" OR "mortality" OR"morbidity" OR "QoL" OR "quality of life") AND (a) AND (e) |
|  | **Secondary aim 3:** To make preliminary assessments of the effects of biomarker testing on costs and mortality associated with the disease. | ("clinical diagnosis” OR “treatment” OR “memory clinic”) AND ("benefit" OR "compliance" OR "mortality" OR" morbidity" OR "QoL" OR "quality of life"AND (a) AND (e) |
|  | **Secondary aim 4:** To monitor disease occurring clinically but not detected by the biomarker testing protocol. | ("clinical diagnosis” OR “memory clinic” OR “criteria” OR "recommendation") AND ("accuracy" OR "sensitivity" OR "specificity" OR"ROC" OR "predictive value" OR "concordance" OR "confirm" OR "negative detection rate" OR "negative referral rate" OR "false negative rate") AND (a) AND (e) |
| **Phase 5: Disease Control Studies** | **Primary aim:** To estimate the reductions in disease-associated mortality, morbidity, and disability afforded by biomarker testing. | ("diagnosis" OR "detection") AND ("benefit" OR "compliance" OR "mortality" OR "morbidity" OR "QoL" OR "quality of life" OR "financial impact" OR "cost" OR "effectiveness") AND (a) AND (e) |
|  | **Secondary aim 1:** To obtain information about the costs of biomarker testing and treatment and the cost per life saved or per quality-adjusted life year | ("diagnosis" OR "detection") AND ("benefit" OR "compliance" OR "mortality" OR "morbidity" OR "QoL" OR "quality of life" OR "financial impact" OR "cost" OR "effectiveness")AND (a) AND (c) AND (e) |
|  | **Secondary aim 2:** To evaluate compliance with testing and work-up in a diverse range of settings. | ("diagnosis" OR "detection") AND ("benefit" OR "compliance" OR "mortality" OR "morbidity" OR "QoL" OR "quality of life" OR "financial impact" OR "cost" OR "effectiveness") AND (a) AND (e) |
|  | **Secondary aim 3:** To compare different biomarker testing protocols and/or to compare different approaches to treating test positive subjects in regard to effects on mortality and costs. | ("diagnosis" OR "treatment") AND ("protocol" OR "recommendation" OR "criteria") AND ("benefit" OR "compliance" OR "mortality" OR "morbidity" OR "QoL" OR "quality of life") AND ("financial impact" OR "cost" OR "effectiveness") AND (a) AND (e) |

1. (“Alzheimer*”)
2. ("Healthy Controls" OR "Cognitively normal" OR "controls" OR "normal").
3. (“MCI” OR “mild cognitive impairment” OR”prodromal”)
4. (other disease/e.g. DLB – if pertinent)
5. (“T807” OR “AV1451” OR “AV-1451” OR “flortaucipir”)
